# The HUNT study: participation is associated with survival and depends on socioeconomic status, diseases and symptoms

**DOI:** 10.1186/1471-2288-12-143

**Published:** 2012-09-14

**Authors:** Arnulf Langhammer, Steinar Krokstad, Pål Romundstad, Jon Heggland, Jostein Holmen

**Affiliations:** 1 HUNT Research Centre, Department of Public Health and General Practice, Norwegian University of Science and Technology, Levanger, Norway; 2 Department of Public Health and General Practice, Norwegian University of Science and Technology, Trondheim, Norway

**Keywords:** Epidemiologic studies, Participation rate, Validity, Selection bias, Mortality

## Abstract

**Background:**

Population based studies are important for prevalence, incidence and association studies, but their external validity might be threatened by decreasing participation rates. The 50 807 participants in the third survey of the HUNT Study (HUNT3, 2006-08), represented 54% of the invited, necessitating a nonparticipation study.

**Methods:**

Questionnaire data from HUNT3 were compared with data collected from several sources: a short questionnaire to nonparticipants, anonymous data on specific diagnoses and prescribed medication extracted from randomly selected general practices, registry data from Statistics Norway on socioeconomic factors and mortality, and from the Norwegian Prescription Database on drug consumption.

**Results:**

Participation rates for HUNT3 depended on age, sex and type of symptoms and diseases, but only small changes were found in the overall prevalence estimates when including data from 6922 nonparticipants. Among nonparticipants, the prevalences of cardiovascular diseases, diabetes mellitus and psychiatric disorders were higher both in nonparticipant data and data extracted from general practice, compared to that reported by participants, whilst the opposite pattern was found, at least among persons younger than 80 years, for urine incontinence, musculoskeletal pain and headache. Registry data showed that the nonparticipants had lower socioeconomic status and a higher mortality than participants.

**Conclusion:**

Nonparticipants had lower socioeconomic status, higher mortality and showed higher prevalences of several chronic diseases, whilst opposite patterns were found for common problems like musculoskeletal pain, urine incontinence and headache. The impact on associations should be analyzed for each diagnosis, and data making such analyses possible are provided in the present paper.

## Background

Population based studies are pivotal for knowledge on health related behavior, prevalence and incidence of symptoms, diseases and death, as well as for assessing potential causal associations in the population. The validity of incidence and prevalence studies rely on the sample of study participants being representative of the actual population, and if participation is influenced by exposures and diseases under study, this may also bias association studies.

Participation rates in population-based studies have declined during the last three decades [1], and this is the case also in most Norwegian epidemiological studies such as the Nord-Trøndelag Health Study (HUNT) [2-4], the Hordaland Health Study [5] and The Oslo Health Study [6].

The adult population of the Nord-Trøndelag County, Norway, has been invited to three main surveys, HUNT1 (1984-86), HUNT2 (1995-97) and HUNT3 (2006-08). Data from this study are extensively used in national and international research, and have per 2012 been the basis of about 600 peer reviewed papers and about 70 doctoral theses. Studies after HUNT1 and 2 indicated only minor potential nonparticipation bias, but these were restricted to few topics [2,7]. Due to a substantial decline in participation rate from 88% in HUNT1, 71% in HUNT2 to 54% in HUNT3, an updated thorough nonparticipation study was warranted.The present study had three main objectives:

a) To study potential participation bias for common symptoms, diseases and socioeconomic status in HUNT3

b) To study mortality by participation status in HUNT 

c) To provide dcata as basis for future sensitivity analyses in disease specific studies

## Methods

### The HUNT study

2In each survey of the adult part of the HUNT Study every citizen of Nord-Trøndelag County aged 20 years and older was invited. The population is homogenous, lives in mainly rural areas with five smaller towns, and has a level of education and income a little beneath the national average. However, mortality and health status is fairly representative of Norway [3,8]. The design and methods applied in the three surveys were by and large unchanged, but each time the scientific program was extended [4].

### Data sources

1) The HUNT3 questionnaires. A questionnaire (Q1) was mailed together with the personal invitation, to be completed and delivered when attending the examination stations. At the examination, questionnaire 2 (Q2) and 3 (Q3) were handed out, these should be completed at home and posted to HUNT Research Centre in a prepaid envelope. Q1 and Q2 included questions about quality of life, life style, symptoms, prior or current diseases and health care utilization, while Q3 (9 versions) was aimed at subgroups with specific diseases and use of health care. Some 99.7% of those delivering Q1 did also participate in the standard examination programme. Nonparticipants received one reminder.

2) Questionnaire for nonparticipants (QNP). About nine months after completion of the HUNT3 survey, a two page questionnaire (QNP) and a prepaid envelope for return were mailed to all nonparticipants in HUNT3 if they were still alive and residing in the county. The questionnaire included identical core questions on symptoms, diseases and life style as in Q1 and Q2, as well as questions on reasons for nonparticipation in HUNT3.

3) General practitioner (GP) data. In 2007 a total of 102 GPs were practising in Nord-Trøndelag County, out of which 2/3 used the electronic patient journal Profdoc, Winmed version 2.2. Among Winmed users, eight practices with 30 GPs were randomly selected for data extraction of selected anonymous data from the patient records during January-May 2011. From patients on the doctors list aged 20-100 by the 1^st^ of January 2011 these data were extracted: Number of consultations during the last year, diagnoses according to The International Classification of Primary Care codes (ICPC2), prescription of selected drugs in the last year and asthma medication during the last five years according to Anatomical Therapeutic Chemical Classification System (ATC).

4) Register data. Data on dispensed drugs during 2008 for inhabitants of the Nord-Trøndelag County was retrieved from the Norwegian Prescription Database (NorPD) [9]. The invitation file for HUNT 3 including variables for age, sex, and participation status was sent to Statistics Norway [10] for merging with data on mortality, education, income (quintiles), social security and social assistance (sick leave, rehabilitation, time-limited or permanent disability pension), marital status and type of municipality. Data comparisons between participants and nonparticipants were performed in an anonymous file. Education, income and marital status were chosen as status of 1^st^ January 2007, whilst the other variables reflected the study period 2006-2008.

### Statistical analyses

In all study parts the numbers of invited and participating individuals are reported, providing several participation rates as recommended [1]. The participation rates derived from questionnaire data refer to Q1 and QNP. Questions derived from Q2 are marked in the tables. When comparing data from Q1 or Q2, QNP and the extracted GP data, results are stratified by age groups (20-39, 40-59, 60-79 and 80+). In the registry and mortality studies we stratified age into three groups, 20-39, 40-64 and 65+, the latter group mainly being retired. For comparison with extracted GP data, only HUNT3 data derived from the corresponding eight municipalities were used. Data on dispensed drugs from NorPD refers to the entire county for the year 2008.

The statistical software SPSS version 17 was used for testing differences between proportions with chi square tests, whilst Stata version 11 for Windows was used for estimation of relative risks with binomial regression (generalized linear model with a log link function assuming a negative binomial distribution), for the estimation of hazard ratios of death in Cox proportional regression analyses, and for age adjusted Kaplan Meier plots of survival. In these analyses follow-up time was from August 1986 for HUNT1, August 1997 for HUNT2 and August 2008 for HUNT3 to the date of death or until 4^th^ of July 2010, whichever came first. Start of follow-up was set to two months after the HUNT surveys to avoid the impact of terminally ill subjects during the surveys.

### Variables

When comparing Q1/Q2 and QNP (Table 1) answers to the question “How is your health at present?” were dichotomized into poor/very poor and good/very good. The mean score of CONOR Mental health Index (MHI) [11], including 7 items each with score 1-4 on mental distress was calculated, and proportions of those scoring above 2.15 are reported. Comparing answers in Q1/Q2 and GP data, combined diagnosis according to ICPC-2 or ATC-codes for medications are reported (Table 2). Anxiety and depressive symptoms were ascertained using the Hospital Anxiety and Depression Score (HADS) [12] in HUNT3, and categorized as dichotomous variables using a cut-off score of ≥ 8 on the HADS-anxiety or HADS-depression, respectively [13].

**Table 1:** Comparisons of anthropometrics (means) and percentages reporting symptoms and diseases between responders to the main questionnaires (Q1 or Q2) and the nonparticipation questionnaire (QNP)

	**Women**					**Men**			
	**Q1**	**QNP**	**p**	**Q1 + QNP**	**Q1**	**QNP**	**p**	**Q1 + QNP**	
Number invited ¤	47 293	19 004			46 567	23020		
Number participated	27 758	3241		30 999	23 049	3677		26 726
Percent of invited to HUNT3	58.7	6.9		65.6	49.5	7.9		57.4
Height (cm) ^#^	164.6	166.3	<0.01	164.7	177.8	179.6	<0.01	178.0
Weight (kg) ^#^	72.9	71.5	<0.01	72.8	86.9	86.7	0.51	86.8
BMI (kg/m^2^) ^#^	26.9	25.8	<0.01	26.8	27.5	26.9	<0.01	27.4
General practitioner last 12 months	83.9	84.6	0.30	83.9	74.5	74.2	0.74	74.4
Hospitalized last year	12.4	20.1	<0.01	13.1	10.9	14.0	<0.01	11.3
Current health poor or very poor	28.4	29.6	0.20	28.5	23.4	24.1	0.36	23.5
Mental distress £	7.9	12.1	<0.01	8.3	6.3	9.4	<0.01	6.8
Insomnia many evenings a week	14.8	16.2	0.03	15.0	7.5	10.2	<0.01	8.0
Wake up early in the morning many days a week	12.2	11.9	0.66	12.1	9.6	8.2	<0.01	9.4
Chronic disease limiting daily functions	33.9	33.9	1.00	33.9	32.4	27.9	<0.01	31.8
Daily cough in periods (Q2)	19.1	20.7	0.04	19.3	22.6	20.3	<0.01	22.2
Attacks of wheezing or breathlessness	12.4	11.3	0.09	12.3	12.3	12.2	0.95	12.3
Allergic rhinitis (Q2)	23.1	25.3	<0.01	23.4	18.7	21.8	<0.01	19.2
Heartburn (a lot) (Q2)	7.1	4.4	<0.01	6.7	7.2	4.8	<0.01	6.8
Headache (Q2)	42.1	44.7	<0.01	42.4	27.8	27.3	0.51	27.7
Migraine (Q2)	10.9	11.8	0.09	11.0	5.0	5.3	0.45	5.0
Musculoskeletal pain of more than 3 months	54.6	41.9	<0.01	53.0	45.1	31.6	<0.01	42.9
Urine incontinence (Q2)	26.8	20.6	<0.01	26.1	8.7	5.2	<0.01	8.2
Medication for arterial hypertension	20.5	24.9	<0.01	20.8	21.4	23.8	<0.01	21.7
Myocardial infarction	1.6	3.1	<0.01	1.7	5.2	6.9	<0.01	5.4
Angina pectoris	2.5	3.2	0.04	2.7	4.8	5.9	0.01	5.0
Cerebral insult	2.2	3.5	<0.01	2.3	3.0	3.7	0.03	3.1
Renal disease	2.6	3.9	<0.01	2.7	2.5	3.8	<0.01	2.7
Asthma	10.4	10.9	0.39	10.4	9.4	10.5	0.04	9.5
COPD or chronic bronchitis	3.3	3.9	0.14	3.3	3.4	5.3	<0.01	3.7
Diabetes	3.8	5.7	<0.01	4.0	4.9	6.6	<0.01	5.2
Cancer	5.6	5.6	0.94	5.6	4.9	4.6	0.55	4.8
Osteoporosis	5.3	5.9	0.20	5.3	0.7	1.1	0.03	0.8
Fibromyalgia	6.3	7.4	0.03	6.4	0.8	1.3	<0.01	0.9
Arthrosis	18.8	21.4	<0.01	19.0	10.3	11.9	0.01	10.5
Sought help for mental problem	16.9	20.6	<0.01	17.7	9.9	11.7	<0.01	10.1
Hyperthyreosis	2.8	3.0	0.56	2.8	0.9	0.5	0.05	0.8
Hypothyreosis	9.3	10.0	0.30	9.4	2.3	1.8	0.06	2.2
Never-smoker §	47.3	44.4	<0.01	47.0	44.3	41.1	<0.01	43.8
Ex-smoker	27.4	26.1	0.13	27.3	35.2	31.1	<0.01	34.6
Occasional smoker	9.4	9.3	0.92	9.4	9.7	9.2	0.38	9.6
Daily smoker	20.6	20.2	0.59	20.6	16.9	18.7	<0.01	17.1
Daily use of snuff	1.2	1.9	<0.01	1.3	13.8	17.0	<0.01	14.3
Alcohol > 2-3 times a week	12.0	9.5	<0.01	11.3	18.8	17.8	0.16	18.6
Exercise > 2-3 times a week	20.5	14.0	<0.01	19.8	17.3	13.9	<0.01	16.9

Questions from Q2 are marked.

¤ Invited to QNP, persons having died or emigrated between HUNT3 and QNP are excluded.

^#^ Height and weight measured at the screening stations, self-reported in QNP.

§ Questions on tobacco smoking; included in n if answered at least one of the smoking related questions.

£ CONOR Mental Health Index (CONOR MHI) consists of 7 questions on mental distress with score 1-4. Mean score calculated and cut-off for dichotomization was ≥ 2.15.

Corresponding data stratified by age and sex are given in Table S1 and Table S2.

**Table 2:** Number of persons, mean age and percentages having consulted general practitioner in the last 12 months among those having answered HUNT3 questionnaire Q1 and those being registered at the general practices

**Characteristics**	**Women age groups**				**Men age groups**			
	**20-39**	**40-59**	**60-79**	**80 +**	**20-39**	**40-59**	**60-79**	**80 +**
Numbers								
HUNT3: Invited	8606	10 577	6274	1400	8528	10 093	6537	2299
HUNT3: Answered Q1	2785	5657	4057	552	3898	6510	4631	782
GPs: Listed patients aged ≥ 20 years	4364	5346	3926	818	3848	5209	3559	1205
Mean age								
HUNT3: Mean age within age groups among all invited	29.7	49.4	67.7	84.4	29.7	49.4	68.1	85.3
HUNT3: Mean age within age groups among all Q1 part.	31.4	50.5	68.0	83.8	31.2	50.3	68.2	84.2
GPs: Mean age within age groups in the patient population	29.0	49.4	67.6	84.8	29.4	49.2	68.0	85.7
Consultations								
HUNT3 Reported GP visit last year	65.0	71.7	86.3	93.7	82.7	82.3	89.7	92.3
GP: Registered > 1 visit to GP last year	58.9	71.3	86.5	92.7	78.9	84.5	90.2	89.2

Data restricted to the municipalities including eight randomly selected general practices (GPs). The 28 872 HUNT3 participants represented 57% of invited in these municipalities.

### Ethics

All participants at HUNT3 were informed and gave written consents to participation in the main and follow-up studies. The Norwegian Data Inspectorate has licensed HUNT Research Centre to store and link data collected in all HUNT surveys. All HUNT surveys, and the present nonparticipant study, are approved by The Regional Committee for Medical and Health Research Ethics.

## Results

In HUNT3, 46 567 men and 47 293 women aged 20 years or more were invited and 23 049 (49.5%) men and 27 758 (58.7%) women answered at least one question in Q1 (Table 1). The lowest participation rates were found in age groups 20-39 and 80+. The questions were completed by 95-99% of the participants with minor differences between diseases, symptoms and life style factors. For life style questions (use of alcohol, snuff, smoking and exercise) the proportion of missing increased by age group from about 2% in the youngest to 12% among those aged 80 years or more. The first question in Q1 was answered by 97.0% whilst the last one was answered by 98.6%. The response rate to questions in Q2 varied from 73% to 80% out of all who answered Q1.

### Comparing HUNT3 questionnaires with nonparticipation questionnaire data

In all 3241 women and 3677 men returned QNP and had answered at least one question. They represent 6.9% and 7.9% of women and men invited to HUNT3 (Table 1). The percentages were similar after exclusion of 498 women and 531 men having died or emigrated between the invitation to HUNT Q1 and QNP. For questions on life style factors missing increased from 1% to 4% by increasing age group, for cardiovascular diseases there were 50% missing data in the youngest age groups. For other diseases and symptoms the proportion of missing increased from 2% in the youngest group to 10-14% in the oldest. Among participants at HUNT2 who were invited to HUNT3, some 70.7% answered Q1 (HUNT3) and 6.4% QNP compared to 29.3% and 8.5% among nonparticipants at HUNT2.

Self-reported body heights and weights were slightly higher and lower, respectively, in QNP compared to measurements of participants at the examination, giving BMI based on measurement 0.6 and 1.1 kg/m^2^ higher in men and women. Except for age group 40-59 years, similar proportions in the two groups had visited GP during the last 12 months, but independent of age and sex, larger proportions in the QNP group had been hospitalized compared to the Q1 group.

More participants of QNP compared to Q1 reported poor/very poor health, mental distress and insomnia in the evenings (Table 1). In women 40 years and older chronic disease limiting daily functions was reported more often in the QNP than Q1 group. No difference between groups were found in men at similar age, but in younger men the highest proportion of affirmative answers to this question was found in the Q1 group.

The prevalence of self-reported symptoms varied between the questionnaires by symptom types. A lot of heartburn was more often reported in Q1/Q2 than in QNP in all age groups, and similar patterns were found for symptoms like chronic musculoskeletal pain and urine incontinence in the age group 20-60 years, but with opposite pattern among the oldest (Table 1, Additional file 1 Table S1, Additional file 2 Table S2). For respiratory symptoms (cough, wheezing and breathlessness), headache and migraine there were minor differences without any consistent pattern by sex or age groups.

Compared to Q1, higher proportions in QNP reported chronic diseases such as arterial hypertension (drug treated), myocardial infarction, angina pectoris, stroke, renal disease, diabetes, fibromyalgia, arthrosis and that they ever had sought help for mental problems in age groups 40-59 years and older. Regarding asthma and COPD there were no substantial differences in women, but in men COPD was reported more often in QNP compared to Q1 in all age groups with 55% difference all over. For hypo- and hyperthyroidism and cancer no between groups difference was found.

Among men above age 39 years, daily smoking and use of snuff were more prevalent in QNP compared to Q1, whilst this was not found among women. In both sexes more never-smokers participated in Q1 compared to QNP. Intake of alcohol 2-3 times a week or more, was reported more often in Q1 than QNP in women, whilst no difference was found among men. In Q1 exercising 2-3 times a week or more, was reported more often than in QNP in both sexes.

When combining data from Q1 and QNP the response rate increased to 65.6% and 57.4% out of those invited to HUNT3 for women and men, respectively. For most symptoms and diseases, the prevalence estimates did not change substantially by combining these data sources (Table 1).

### Comparing HUNT3 questionnaire with general practice data

In all, data on 13 821 women and 14 454 men were extracted from GP records. The age and sex distribution of the GP population was representative of the county population (Table 2).

A lot of heartburn was reported less often in HUNT than diagnosed heartburn or esophagitis by GPs (Table 3), and higher prevalence was found for prescriptions of medications mainly used at these indications (data not shown). The proportion of patients with diagnoses of cardiovascular diseases (angina, myocardial infarction and stroke) in GP records was higher than the prevalence of corresponding self-reported diseases. There was, however, close agreement between the two data sets regarding use of antihypertensive treatment. Interestingly, data from NorPD showed more prevalent use of such drugs, but these are also indicated for other diseases than arterial hypertension. In HUNT3 there were questions on each of the diagnoses of spondylarthritis and rheumatoid arthritis, whilst these diseases have a common diagnostic code in ICPC-2. Combining these diagnoses showed a fair agreement between the two sources, and similar consistency was also found for arthrosis and osteoporosis in women, but GP-data indicated 20% higher prevalence of arthrosis among men than reported by HUNT participants.

**Table 3:** Prevalence data (%) based on HUNT3 questionnaires (Q1 or Q2) and general practitioner diagnoses (GPD)

	**Women age groups**				**Men age groups**			
	**20-39**	**40-59**	**60-79**	**80 +**	**20-39**	**40-59**	**60-79**	**80 +**
**Heart burn**								
Q2: A lot of heart burn	6.1	7.9	6.3	6.2	4.9	6.3	8.6	7.9
GPD: Heart burn or reflux (D03/D84)	3.9	8.2	10.0	11.0	2.5	7.6	12.1	12.0
**Cardiovascular diseases**								
Q1: Angina pectoris	0.3	1.5	9.6	21.4	0.2	0.7	4.4	15.5
GPD: Angina pectoris (K74)	0.1	2.2	13.9	26.4	0	0.8	6.8	21.1
Q1: Myocardial infarction	0.1	2.3	10.1	21.2	0.0	0.5	3.0	8.6
GPD: Myocardial infarction (K75or K76)	1.0	3.3	12.9	18.8	0.5	1.3	4.7	10.4
Q1: Cerebral insult	0.6	1.5	5.4	11.6	0.3	1.1	3.9	8.6
GPD: Cerebral insult (K90 + 91)	0.4	2.2	7.3	17.0	0.4	1.4	5.7	14.2
Q1: Arterial hypertension	1.2	14.7	38.1	44.1	2.1	12.7	36.9	49.8
GPD: Arterial hypertension (K86 or K87)	2.7	14.7	37.4	42.2	2.2	12.9	37.1	49.4
NorPD: Drugs arterial hyper-tension (C02-03, 07–09)	1.7	16.0	51.5	70.3	2.2	15.7	49.5	67.7
**Inflammatory and musculoskeletal diseases**								
Q1: Spondylarthritis or								
rheumatoid arthritis	1.5	4.1	5.3	6.0	1.9	4.7	7.2	8.8
GPD: Spondylarthritis or rheumatoid arthritis (L88)	1.5	3.4	4.5	5.6	1.5	4.7	7.9	8.7
Q1: Arthrosis	0.8	6.1	17.2	23.3	1.0	12.8	34.2	45.5
GPD: Arthrosis hip or knee (L 89 + 90 + 91)	1.7	7.5	21.5	29.8	1.7	10.2	33.6	43.4
Q1: Osteoporosis	0.1	0.3	1.2	3.1	0.3	1.9	11.1	20.6
GPD: Osteoporosis L95	0.2	0.6	1.5	2.9	0.2	1.4	10.8	22.7
**Neurological diseases**								
Q1: Headache	37.0	34.3	18.7	10.7	56.9	47.7	26.8	16.6
GPD: Headache (N01)	10.8	9.9	7.9	6.1	18.6	16.5	12.6	11.0
Q1: Migraine	3.1	4.5	2.7	3.4	10.4	11.2	5.1	5.0
GPD: Migraine (N89)	3.8	4.4	2.6	1.5	9.5	11.6	7.1	2.4
NorPD: Use of migraine drugs (N02)	1.0	1.0	0.5	0.2	3.5	4.9	2.0	0.3
Q1: Epilepsy	1.5	1.2	1.2	1.9	1.3	1.4	0.7	1.4
GPD: Epilepsy (N88)	1.8	1.7	1.8	1.5	1.5	1.7	1.5	1.2
**Mental diseases and insomnia**								
Q2: HADS anxiety score > 8#	11.9	11.7	7.2	8.2	18.4	17.4	15.6	13.2
GPD: Anxiety or feeling of anxiety (P 01 + 74 +79)	6.7	8.5	8.5	8.6	9.7	14.1	14.0	15.4
Q2: HADS depression score > 8#	6.4	9.4	11.7	18.8	6.2	8.3	9.8	16.3
GPD: Depression or feeling of depression (P 03 + 76)	11.0	18.1	16.0	11.1	19.6	31.3	28.0	21.7
Q2: Insomnia many evenings or mornings a week	11.6	13.8	16.2	15.4	13.1	21.3	25.8	25.3
GPD: Sleeping disorder (P06)	6.3	8.6	18.8	24.0	6.3	13.3	21.7	32.4
Q1: COPD or chronic bronch	1.0	2.0	5.6	5.4	1.5	2.7	4.6	4.2
GPD: COPD or chronic bronchitis (R95)	0.1	1.9	7.9	13.2	0.2	1.8	7.2	7.0
Q1: Asthma	10.5	9.3	9.4	7.2	12.4	9.4	10.4	8.6
GPD: Asthma (R96)	10.6	7.0	9.2	10.0	10.4	9.7	11.9	9.9
Q1: Use of asthma or copd drugs in the last 5 years	8.1	7.5	11.3	9.6	10.4	9.6	12.5	9.7
GP prescription asthma drugs in the last 5 year (R03)	7.4	8.7	15.1	17.4	9.8	11.4	17.3	12.6
NorPD: Use of asthma drugs in 2008 (R03)	4.6	5.6	11.6	14.2	5.7	7.7	13.0	9.1
**Skin diseases**								
Q1: Psoriasis	4.1	6.8	7.0	3.8	3.3	6.2	6.6	4.1
GPD: Psoriasis (S91)	2.0	4.6	5.5	4.6	2.7	4.5	5.8	2.8
**Endocrine disorders**								
Q1: Hyperthyreosis	0.3	0.7	1.3	2.5	1.4	2.6	3.4	3.2
GPD: Hyperthyreosis (T85)	0.2	0.5	0.7	0.7	0.6	1.9	2.0	2.0
Q1: Hypothyreosis	0.6	2.1	2.9	5.3	4.1	8.0	12.3	12.1
GPD: Hypothyreosis (T86)	1.4	1.9	4.2	6.7	3.2	7.8	12.7	13.8
GP prescription thyroid hormone (H03AA01)	0.5	1.5	3.3	5.1	2.2	7.0	12.1	12.5
NorPD: Use of thyroid hormone (H03AA01)	0.5	2.0	3.7	5.9	2.6	7.9	14.2	13.4
Q1: Diabetes mellitus	0.8	3.4	9.2	9.2	0.8	2.4	6.9	9.7
GPD: Diabetes mellitus (T89 + 90)	1.2	4.8	13.7	16.3	1.4	3.6	10.4	13.0
GP prescription antidiabetic drug (A10A or A10B)	0.8	4.0	10.9	10.9	1.2	2.8	7.9	8.8
NorPD: Antidiabetic drug (A10A or A10B)	1.1	4.1	11.1	11.7	1.5	2.6	8.4	9.2
**Urinary tract**								
Q2: Urinary incontinence	4.7	7.6	10.4	13.1	22.2	27.5	27.4	32.8
GPD: Urinary incontinence (U04)	1.0	0.5	3.0	8.7	2.2	6.8	10.5	22.0

HUNT data are restricted to the municipalities being represented by eight randomly selected general practices, and include 28 872 HUNT3 participants (57% of invited in these municipalities). Data from Norwegian Prescription Registry (NorPD) include the entire county for the year 2008.

# Hospital Anxiety and Depression Scale (HADS) 14 items, cut-off 8 indicate minor, moderate and severe disease (REF).

In parenthesis: For diagnosis – codes according to the International Classification of Primary Care (ICPC2), for medication codes according to The Anatomical Therapeutic Chemical Classification System (ATC).

A high proportion of participants in HUNT3 reported bothersome headache, but the prevalence of the diagnosis of headache in GP records was close to one third of this. However, for the more specific diagnosis of migraine, the prevalence in general practice was only slightly lower than self-reported prevalence in HUNT3. The frequency of migraine attacks varies from a few attacks per decade to many attacks a week; in line with this NorPD data showed that only 0.8% of men and 3.4% of women had migraine medication dispensed during one year (2008).

Data in HUNT3 were not eligible for direct comparison of prevalence of anxiety and depression with the GP data. However, the prevalence of diagnosed anxiety by GP was slightly lower compared to the prevalence having a HADS-anxiety symptom score of 8 or higher. Corresponding comparison between diagnosed depression and HADS depression symptom score 8 or higher showed much lower estimated prevalence of depression amongst HUNT3 participants. Among the oldest age groups sleeping disorders were more often diagnosed in GP records than reported by HUNT participants, whilst the opposite pattern was found in younger age groups.

Higher prevalence of COPD diagnosis was found in GP data compared to self-reported data in HUNT3 among those aged 60 years and older, whilst there were relatively small differences regarding asthma. Reported use of asthma or COPD medication in HUNT3 was somewhat lower compared to data from GP, especially in the older age groups.

For hypothyreosis and corresponding prescribed and dispensed thyroid hormones there were rather consistent data, whilst hyperthyreosis was less often diagnosed in GP records. The diagnosis of diabetes was more prevalent in the GP population than the self-reported data from HUNT3 indicated. Data on both prescribed and dispensed insulin and oral medication for diabetes suggested an underestimation of prevalence data from HUNT3, as a substantial number with diabetes also are treated with life style only. Self-reported urine incontinence was more prevalent in HUNT3 compared to the proportion having been diagnosed by GPs.

### Comparing data from GP records, questionnaire Q1 and questionnaire QNP

**Figure 1:**
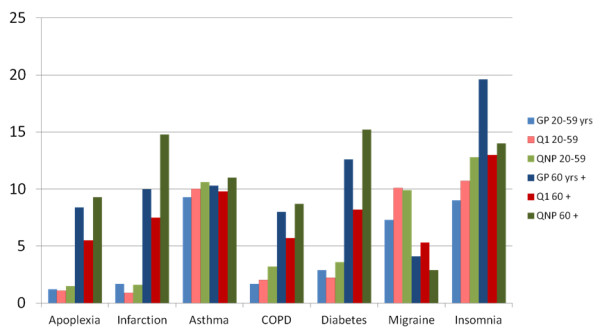
Prevalence of diseases (%) based on GP records, HUNT questionnaire 1 (Q1) and QNP by age group.

Comparing over all prevalence either of diagnoses registered in general practices and self-reported data from Q1 and Q2 show minor differences for asthma (Figure 1). For persons 60 years and older, there were similar patterns for apoplexia, myocardial infarction, COPD and diabetes, with lowest prevalence in Q1, intermediate in GP-data and highest among QNP-participants. Assuming that the prevalences of chronic diseases found in the QNP group were representative for the entire nonparticipation group 60 years and older, the combined prevalences for nonparticipants and Q1 participants compared to estimates from GP records, would be 6.6 *versus* 8.4% for apoplexia, 9.8 *versus* 10.0% for myocardial infarction, 9.9 *versus* 10.3% for asthma, 5.7 *versus* 8.0% for COPD and 10.3 *versus* 12.6% for diabetes.

Among persons younger than 60 years, symptomatic diseases like COPD, migraine and insomnia were more prevalent in Q1 and QNP data than diagnosed in general practice.

### Self-reported reasons for nonparticipation

About 10% reported that they had not received invitation to HUNT3 (Table 4). Otherwise the most important reason for nonparticipation was lack of time or inconvenient session, this was reported by 50.7% in women and 56.6% in men. Among the two oldest age groups, 4.0 % of women and 6.4% of men reported they would have no benefit of such examinations, while 11.7% of women and 7.7% of men reported being too ill to participate.

**Table 4:** Percentages reporting reasons for nonparticipation in the HUNT study among 3241 women (W) and 3677 men (M) having answered the nonparticipation questionnaire (QNP)

**Reasons**	**20-39 years**		**40-59 years**		**60-79 years**		**80 + years**		**All age groups**		
	**W**	**M**	**W**	**M**	**W**	**M**	**W**	**M**	**W**	**M**	**p**^**§**^
Did not receive the invitation	13.3	14.9	6.2	9.3	8.5	9.1	8.0	10.7	9.5	11.1	0.02
Had not time /inconvenient session	60.7	64.5	54.9	61.9	34.8	40.2	14.3	20.8	50.7	56.6	<0.01
Don’t rely on such examinations	0.2	0.9	1.1	1.6	1.7	2.2	0.4	2.0	0.8	1.5	<0.01
No benefit of such examinations	0.3	1.7	1.6	1.8	3.1	4.8	6.3	8.1	1.7	2.6	<0.01
Too ill to attend the study	1.4	0.6	3.3	1.2	7.2	5.5	23.7	19.5	4.7	2.6	<0.01
Questions I did not wish to answer	2.1	1.2	3.9	3.5	6.8	7.0	3.6	4.0	3.7	3.5	0.61
Other reason	25.1	17.5	30.0	22.1	32.1	27.2	24.6	18.8	28.1	21.5	<0.01

^§^ p-values for chi-square tests comparing all men and women.

### Comparing participants and non-participants in national register data

The anonymous file included data on a) participants (27 758 women and 23 049 men), b) those having answered QNP (3241 women and 3677 men) and c) those with no participation (16 294 women and 19 841 men). The analyses include group “a” and “c”. Participation in HUNT depended on sex, age, marital status, education and income (Table 5). In those who received disability pension the participation was lower, and even lower in those receiving social benefit compared to those not receiving social security money. The participation was lower in urban compared to rural municipalities.

**Table 5:** Prevalence ratio and absolute difference (%) of participation (n = 50 807)*versus*no participation*(n = 36 135) in the HUNT3 main questionnaire by different characteristics

**Characteristic**	**Prevalence ratio**			**Difference in participation (%)**		
	**Crude**	**Adjusted**	**95% CI**	**Crude**	**Adjusted**	**95% CI (%)**
Sex (women/men)	1.17	1.23	1.20, 1.26	9.2	12.6	11.8,13.3
20-39 year	1	1	Reference	0	0	Reference
40-59 year	1.63	1.45	1.40, 1.50	25.2	19.1	18.2, 20.0
60-79 year	1.88	1.74	1.68, 1.81	34.7	29.9	29.0, 30.8
80 + yrs	1.12	1.23	1.16, 1.30	4.8	10.7	9.2, 12.1
**Education**						
9 year or less	1	1	Reference	0	0	Reference
10-12 year	1.28	1.27	1.23, 1.30	13.6	13.6	12.8, 14.5
University	1.39	1.36	1.31, 1.41	19.0	17.4	16.4, 18.4
**Marital status**						
Unmarried	1	1	Reference	0	0	Reference
Divorced/Widower	1.27	1.01	0.97, 1.05	11.6	−1.8	−3.0, -0.6
Married/cohabits	1.64	1.29	1.25, 1.33	27.3	14.5	13.5, 15.4
**Income**						
Quintile 1	1	1	Reference	0	0	Reference
Quintile 3	1.39	1.32	1.09, 1.60	15.9	15.1	10.0, 20.1
Quintile 5	1.49	1.42	1.17, 1.73	20.4	20.4	15.6, 25.1
**Municipality**						
Urban / rural	0.93	0.91	0.89, 0.93	6.1	−5.6	−6.4, -4.7
**Social security #**						
Disability pension	0.97	0.92	0.88, 0.96	−19.2	−6.5	−7.8, -5.2
Social benefit	0.43	0.61	0.56, 0.67	−32.8	−17.9	−20.2,-15.5

*Participants that answered the nonparticipation questionnaire (QNP) were not included.

¤ Binomial regression, adjusted for all other variables except social security factors.

#Analysis restricted to subjects below 65 years of age and all variables entered in the adjusted model.

**Figure 2:**
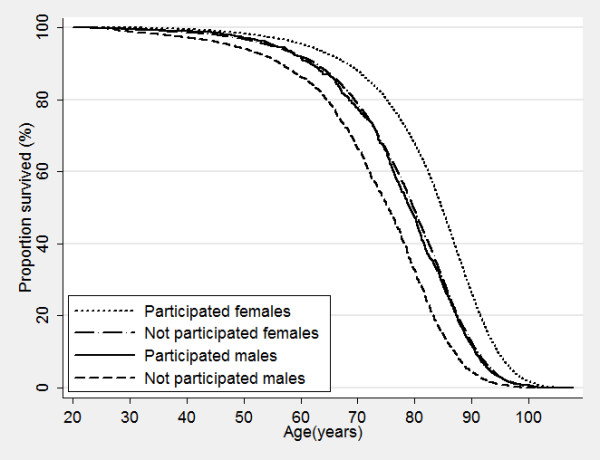
**Overall survival by participation in HUNT 1 in men and women.** Age used as timescale.

**Figure 3:**
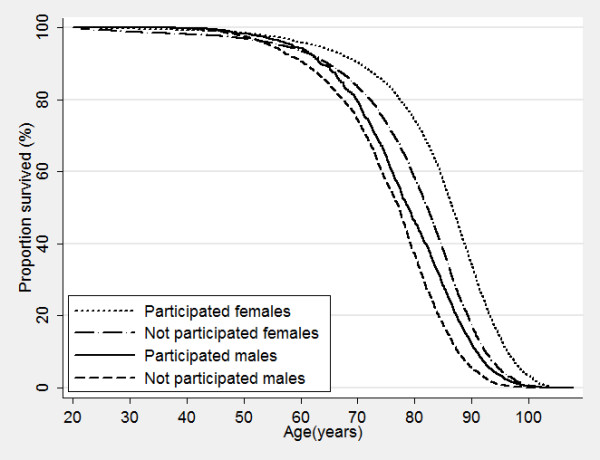
**Overall survival by participation in HUINT 2 in men and women.** Age used as timescale.

**Figure 4:**
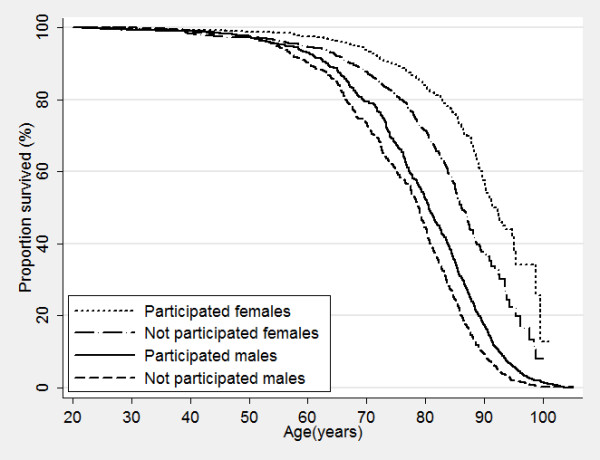
**Overall survival by participation in HUNT 3 in men and women.** Age used as timescale.

**Figure 5:**
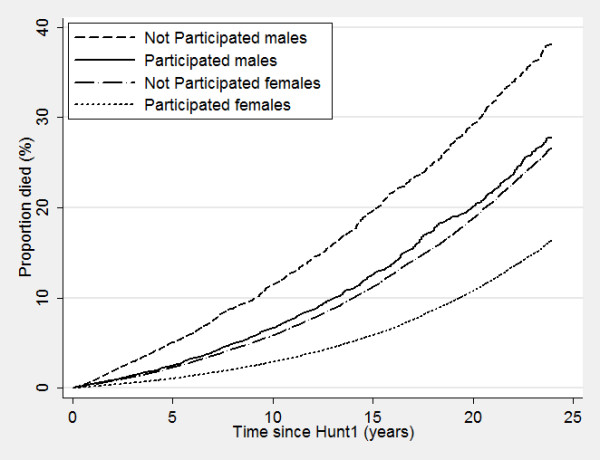
Risk of death by participation in HUNT 1 in men and women adjusted for age (estimated at 50 years of age at participation).

**Figure 6:**
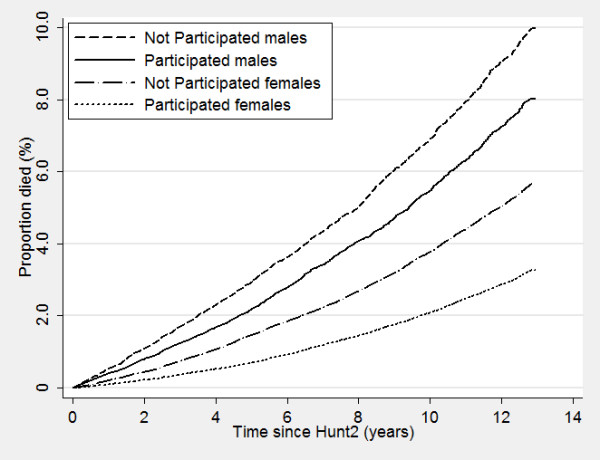
Risk of death by participation in HUNT 2 in men and women adjusted for age (estimated at 50 years of age at participation).

**Figure 7:**
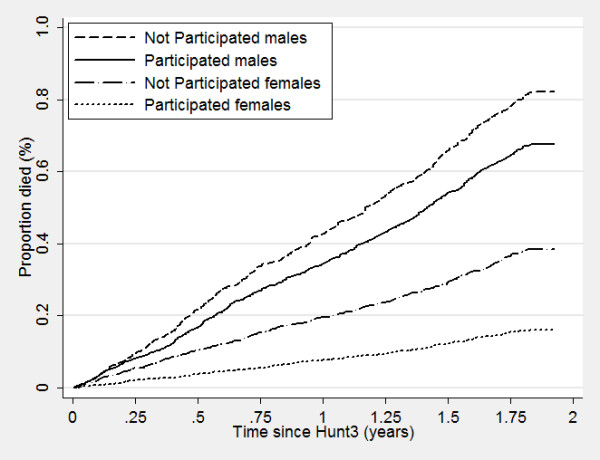
Risk of death by participation in HUNT 3 in men and women adjusted for age (estimated at 50 years of age at participation).

Nonparticipants of HUNT surveys displayed a higher mortality even many years after the surveys took place, with increased mortality of 50%, 72% and 180% for nonparticipants compared to participants in HUNT1, HUNT2 and HUNT3, respectively (Table 6). Figure 2, 3 and 4 show corresponding survival curves of participants and nonparticipants of HUNT1 to 3 using age as the timeline and Figure 5, 6 and 7 show the proportion having died, adjusted for age (at 50 years), by time since the HUNT surveys. Interestingly, there are similar patterns for risk of death between participants and nonparticipants in surveys with participation rate varying from 88 to 54%.

**Table 6:** Overall and sex specific age adjusted mortality (hazard ratio with 95% CI) according to participation in the HUNT 1, HUNT 2 and the HUNT 3 study

**Participation**	**N**	**Deaths**	**Hazard ratio**	**95% CI**
**HUNT 1 (1984–86)**				
Women				
Participated	38698	13450	1.00	Reference
Not participated	3571	1300	1.51	1.43-1.60
Men				
Participated	36928	14206	1.00	Reference
Not participated	5018	1391	1.55	1.46-1.64
Overall				
Participated	75626	27656	1.00	Reference
Not participated	8589	2691	1.50	1.44-1.57
**HUNT 2 (1995–97)**				
Women				
Participated	34469	5087	1.00	Reference
Not participated	9700	2833	1.82	1.74-1.91
Men				
Participated	30237	5497	1.00	Reference
Not participated	12625	2261	1.64	1.56-1.73
Overall				
Participated	64706	10584	1.00	Reference
Not participated	22325	5094	1.72	1.66-1.78
**HUNT 3 (2006–08)**				
Women				
Participated	27652	835	1.00	Reference
Not participated	19405	225	3.33	2.86-3.87
Men				
Participated	22918	367	1.00	Reference
Not participated	23443	702	2.41	2.12-1.75
Overall				
Participated	50571	619	1.00	Reference
Not participated	42848	1555	2.80	2.54-3.09

Adjusted for age by using age as the time scale.

The following subjects are excluded from the analyses: Persons < 20 years of age at baseline study, and deaths between invitation and August 1986, August 1997, and August 2008 for HUNT1, HUNT2 and HUNT3, respectively. The number of participants and nonparticipants therefore are lower than previously for the entire study population.

## Discussion

Participation rates in HUNT3 depended on age, sex, socioeconomic status and type of symptoms and diseases. Among nonparticipants, the prevalence of common chronic diseases was higher compared to that reported by participants. This included cardiovascular diseases and diabetes mellitus, a pattern confirmed by prevalence data based on diagnosis by GPs. Contrary to this, at least among people younger than 80 years, common problems like musculoskeletal pain, urine incontinence and headache were reported more often in participants compared to nonparticipants. The study confirms associations between participation and marital and socioeconomic status, and maintenance of increased risk of death for nonparticipants even many years after the surveys. Combining data from different sources provides the opportunity for future sensitivity analyses of prevalence, incidence and association studies.

Except for the Tromsø Study [14], most national [3,5,6,15] and international studies [1,16,17] have reported corresponding reduction in participation rate as the HUNT study. Reasons for the increase of nonparticipation in population based studies are thoroughly discussed by Galea et al. [1], and may also be relevant to the Norwegian population; People might be overloaded with invitations to research and marketing surveys, there is a general decrease in volunteerism parallel to decrease in participation in organizations and social activities, there might be lack of immediate benefit for the individual participant and, generally, there is an increasing disillusionment with science due to conflicting results between different studies and changing recommendations for behavioral risk factors. Further, more complex procedures regarding informed consent and study protocol as well as the burden of being invited to follow-up studies, might decrease study participation [18,19].

The HUNT surveys have also become more complex and demanding for participants with more comprehensive questionnaires, interviews and examinations. Further, in order to keep to laws and regulations for medical research, an eight pages information folder was sent together with the personal invitation and consent form, even though invited persons prefer more simple forms [18]. Increased number and size of follow-up questionnaires (Q2 and Q3s), however, have not influenced a stable response rate of these of about 75-80% among those having attended the examination stations in three surveys. This indicates that the length and number of questionnaires have minor effect on the all over participation in those who from the start had decided to participate [20-22]. Our data show higher participation rate in HUNT3 among persons previously having participated in HUNT2 compared to nonparticipants, this is in line with results from another Norwegian Cohort Study [23]. Previous participation status in HUNT2 did, however, not influence the participation rate for QNP, indicating similar attitude to contribute in data collection by short questionnaires in participants and nonparticipants.

Low participation rates among persons under the age of 40 probably reflects less opportunity to spend time, limited short time benefit for a rather healthy age group [1,24] and less altruistic attitude in contributing to research [1]. Higher prevalence of chronic diseases having regular follow-up by health care among nonparticipants and opposite pattern for bothersome, but more trivial symptoms in HUNT3, do support potential benefit for the individual person to be of importance when considering participation. With increasing diagnostic and therapeutic opportunities in the health care during the last decades, present surveys are considered less as a supplement to the health care.

The present study confirms previous studies having reported low participation among people who are young, unmarried, and belonging to lower socioeconomic groups [5,6,21,25-28]. Interestingly, also in the HUNT Study persons in higher socioeconomic groups, with presumably highest time pressure, participated more often compared with lower socioeconomic groups, indicating that motivation and attitude to research are important.

About 10% of nonparticipants claimed that they had not received the invitation. We have no indication that this can be explained by failure in the post delivery. However, the invitations were sent in plastic foils, and this could have been taken as advertising leaflets and therefore not read. Economic costs should not have much influence on the participation rate; the participation was free of charge and most people were allowed by employers to meet during working time with full salary. To avoid nonparticipation due to the inconvenience of leaving work, the examination stations were open even in the early evenings. Participation was not stimulated by financial incentives, as previous studies have not found this to increase response rates [20].

There has been a general positive attitude towards health related research in the population of Nord-Trøndelag. Since HUNT2 (1995-97) there have been regular reports from HUNT-related research on the HUNT web-site and in the media. The HUNT Study has been supported by the Norwegian Parliament, the Government, the Ministry of Health, and there has been a close collaboration between the County Council, the municipalities and HUNT Research Centre. Additionally, the media have been strongly supportive of the HUNT Study.

### Influence on incidence and prevalence estimates

Most studies have found little evidence for substantial bias due to nonparticipation [6,20,28-30]. In a Norwegian community respiratory cohort study, increasing the response rate from 65 to 89% after three reminders, resulted in no overt differences in incidence rates of respiratory symptoms and asthma as well as their associations to sex, age and smoking habits [31]. However, like the present study, others have reported underestimation of psychiatric disorders due to nonparticipation [5,32,33]. The scores of HADS anxiety and depression symptoms cannot be directly compared with GP’s diagnosis of anxiety and depression. However, our finding of higher prevalence of reported anxiety symptoms compared to anxiety diagnosis, and lower prevalence of reported depressive symptoms compared to depression diagnosis, in participants compared to the background population, indicates that depression is a more restricting factor for participation than anxiety.

In line with other studies, nonparticipants in HUNT3 was also characterized by more unhealthy lifestyle regarding tobacco smoking and physical inactivity [26,34], and poorer somatic status [25,30,35,36]. Data from the GPs further indicates as much as 50% higher prevalence of angina, myocardial infarction and stroke compared to the HUNT3 data, a pattern also found in other cardiovascular studies [25,35]. Correspondingly, the diabetes prevalence based on HUNT3 Q1 was underestimated both compared to QNP and the GP data based on diagnosis and prescription of medication. Higher prevalence indicated by GP diagnosis than by prescription data could be explained by prescription of insulin by hospital doctors for young adults, and attainment of adequate diabetic control by change in life style for many patients with diabetes type II.

The difference in BMI estimated by self-reported measures compared to measures at the examination stations in HUNT3 is in line with data from 2008 in an Australian study [37]. Lower participation rate among lower socioeconomic groups could contribute to reduce the difference between self-reported and measured anthropometrics.

### Influence on associations

Many studies have found that subjects with risk behavior like smoking, high alcohol consumption or drug use are underrepresented in studies addressing these factors [38,39]. The corresponding pattern in the present study is mainly supposed to be related to differences in participation due to socioeconomic factors, as life style factors by themselves amounted to a small fraction of all questions. Studies on effects of environmental and occupational exposures have experienced participation bias depending on exposures measured [40,41]. By analyzing exposures in blood samples and linking HUNT data to external registers, a lot of associations can be analyzed, but these should not be influenced by participation as these topics have not been focused prior to the surveys.

Nonparticipation bias may be greater in surveys with higher participation rates compared with those with lower participation rates [34], as the differences between participants and nonparticipants may exaggerate real differences between participants and the eligible population sampled [25]. In the present study, however, inclusion of nonparticipants answering QNP increased the prevalence estimates for chronic diseases slightly, but this correction decreased the gap slightly in prevalence between the record data from general practice and HUNT participants. If we assume that the prevalences found among QNP participants 60 years and older, were representative of the entire nonparticipant group, the combined prevalences for the nonparticipants and Q1 participants would be close to the estimates found in the GP population for myocardial infarction and asthma, whilst for COPD, apoplexia and diabetes the GP population would still have higher prevalences. Strategies for improving participation rate should be developed and evaluated prior to the next HUNT survey. In this population increased participation rate seem to improve the external validity of prevalence estimates. After HUNT3 there has been a focus on overweight, obesity and laziness in the media. This might introduce differential influence on participation in future surveys due to stigmatization or victim blaming.

Nonparticipation has been found to be associated with two times higher risk of death independent of socioeconomic category in nonparticipants compared to participants, both in a 20 years follow-up study of white collar workers aged 35-55 years [42] and the FINRISK study, inviting persons aged 35-74 years [43]. Higher mortality risk among nonparticipants could be due to disabling diseases hindering study participation. To avoid inclusion of end stage patients in the present study, start of follow-up after each of the HUNT surveys were set to 2 (or two) months after study inclusion.

Strengths of this study were inclusion of several data sources for evaluation of nonparticipation bias, GP data representing background data regarding diagnoses and use of medication for the entire population, register data revealing potential nonparticipation bias by socioeconomic status and QNP reflecting the nonparticipation group. The representativeness of responders to QNP for the entire nonparticipation group might be questioned, but sensitivity tests for chronic diseases indicate that the validity of these data is rather good.

Limitations of the study were inability to link data from general practices to HUNT-data due to patient confidentiality, use of registered diagnoses in general practices that could lead to some extent of underestimation of prevalence if doctors had not registered diagnoses given by previous GPs, and comparison of chronic diagnoses with symptom report for the last 12 months (for example head ache, urine incontinence). Background data from the GPs were collected three years after HUNT, but this should not introduce important bias in prevalence estimates.

Multiple testing may contribute to statistical significances by chance, but having this in mind, there seem to be consistent results on differences that researchers have to take into consideration.

## Conclusions

A participation rate of 54% in HUNT3 was lower than expected, but for age groups 60-80 years the rates were 65% to 70%. Compared to nonparticipation questionnaire and records from general practice, the prevalences, however, seem to be somewhat underestimated for chronic diseases already being treated in the health care. Higher prevalence among nonparticipants of cardiovascular diseases, diabetes and mental distress seem to parallel differences in socioeconomic groups and risk factor exposure. So far there is no reason to be concerned about introduction of bias in association and causal studies, but inclusion of a wide spectre of data and more sources for background data provide opportunity for addressing this in disease specific sensitivity studies.

## Competing interests

The authors declare that they have no competing interests.

## Authors’ contributions

Langhammer A was the head of the questionnaire group in HUNT 3, and he is head of HUNT Databank. He planned the nonparticipation study, collected data, performed statistical analyses and drafted the manuscript. Krokstad S was the project leader of HUNT3, and he participated in planning, analyzes and commented on the manuscript. Romundstad P contributed in statistical analyses and commented on the manuscript. Heggland J was responsible for technical development of HUNT databank, contributed in data storage and quality assurance and commented on the manuscript. Holmen J was head of HUNT research Centre and the HUNT Study in 1984-2008, and he has commented on the manuscript.

## Authors’ information

None.

## Pre-publication history

The pre-publication history for this paper can be accessed here:

http://www.biomedcentral.com/1471-2288/12/143/prepub

## Supplementary Material

Additional file 1 **Table S1.** Comparisons of anthropometrics (means) and percentages reporting symptoms and diseases between participants having answered questionnaire 1 (Q1) (23 049) or questionnaire 2 (Q2), and those having answered a shortened nonparticipation questionnaire (QNP) (n = 3677) among men stratified by age groups. Click here for file

Additional file 2 **Table S2.** Comparisons of anthropometrics (means) and percentages reporting symptoms and diseases between participants having answered questionnaire 1 (Q1) (27 758) or questionnaire 2 (Q2), and those having answered a shortened nonparticipation questionnaire (QNP) (n = 3241) among women stratified by age groups. Click here for file
