# Supplementary material for: The HUNT study: participation is associated with survival and depends on socioeconomic status, diseases and symptoms
Source: BMC Med Res Methodol. 2012 Sep 14;12:143. doi: 10.1186/1471-2288-12-143 (PMC3512497; doi:10.1186/1471-2288-12-143)
Supplement: Additional file 1 — Table S1. Comparisons of anthropometrics (means) and percentages reporting symptoms and diseases between participants having answered questionnaire 1 (Q1) (23 049) or questionnaire 2 (Q2), and those having answered a shortened nonparticipation questionnaire (QNP) (n = 3677) among men stratified by age groups. [file 1471-2288-12-143-S1.doc]

Table 1a.Comparisons of anthropometrics (means) and percentages reporting symptoms and diseases between participants having answered questionnaire 1 (Q1) (23 049) or questionnaire 2 (Q2), and those having answered a shortened nonparticipation questionnaire (QNP) (n=3677) among men. Questions from Q2 are marked.

|  | Age groups (age at invitation) | | | | | | | | | | | | | | | |
| --- | --- | --- | --- | --- | --- | --- | --- | --- | --- | --- | --- | --- | --- | --- | --- | --- |
| MEN | 20-39 years | | | 40-59 years | | | 60-79 years | | | 80 years + | | | Total | | | |
|  | Q1 | QNP | p | Q1 | QNP | p | Q1 | QNP | p | Q1 | QNP | p | Q1 | QNP | p | Q1+QNP |
|  |  |  |  |  |  |  |  |  |  |  |  |  |  |  |  |  |
| Number invited ¤ | 15 207 | 10 408 |  | 18 110 | 8064 |  | 10 920 | 3431 |  | 2330 | 1117 |  | 46 567 | 23020 |  |  |
| Number participated | 4735 | 1305 |  | 9975 | 1544 |  | 7338 | 700 |  | 1001 | 128 |  | 23 049 | 3677 |  | 26 726 |
| Percent of invited to HUNT3 | 31.1 | 8.6 |  | 55.1 | 8.5 |  | 67.2 | 6.4 |  | 43.0 | 5.5 |  | 49.5 | 7.9 |  | 57.4 |
|  |  |  |  |  |  |  |  |  |  |  |  |  |  |  |  |  |
| Height (cm) ^#^ | 179.9 | 180.8 | <0.01 | 178.8 | 179.9 | <0.01 | 175.7 | 177.3 | <0.01 | 171.8 | 174.5 | <0.01 | 177.8 | 179.6 | <0.01 | 178.0 |
| Weight (kg) ^#^ | 86.4 | 86.3 | 0.92 | 88.9 | 88.6 | 0.54 | 85.6 | 84.6 | 0.08 | 78.3 | 77.0 | 0.25 | 86.9 | 86.7 | 0.51 | 86.8 |
| BMI (kg/m^2^) ^#^ | 26.6 | 26.4 | 0.02 | 27.8 | 27.4 | <0.01 | 27.7 | 26.9 | <0.01 | 26.5 | 25.1 | <0.01 | 27.5 | 26.9 | <0.01 | 27.4 |
|  |  |  |  |  |  |  |  |  |  |  |  |  |  |  |  |  |
| **Health care utilisation** |  |  |  |  |  |  |  |  |  |  |  |  |  |  |  |  |
| General practitioner last 12 months | 63.7 | 66.0 | 013 | 70.2 | 74.8 | <0.01 | 84.9 | 85.6 | 0.70 | 90.9 | 89.3 | 0.62 | 74.5 | 74.2 | 0.74 | 74.4 |
| Hospitalized last 12 months | 6.9 | 8.0 | 0.21 | 9.1 | 11.5 | <0.01 | 14.6 | 27.2 | <0.01 | 20.9 | 36.4 | <0.01 | 10.9 | 14.0 | <0.01 | 11.3 |
|  |  |  |  |  |  |  |  |  |  |  |  |  |  |  |  |  |
| **Self reported health and current symptoms** |  |  |  |  |  |  |  |  |  |  |  |  |  |  |  |  |
| Current health poor or very poor | 10.3 | 12.9 | <0.01 | 20.4 | 23.6 | <0.01 | 32.8 | 40.2 | <0.01 | 47.5 | 58.3 | 0.03 | 23.4 | 24.1 | 0.36 | 23.5 |
| Mental distress £ | 5.9 | 8.5 | <0.01 | 6.9 | 10.0 | <0.01 | 5.7 | 8.9 | <0.01 | 6.8 | 14.4 | 0.01 | 6.3 | 9.4 | <0.01 | 6.8 |
| Insomnia many evenings a week | 9.0 | 9.2 | 0.86 | 7.5 | 11.8 | <0.01 | 6.9 | 7.8 | 0.36 | 7.6 | 15.0 | <0.01 | 7.5 | 10.2 | <0.01 | 8.0 |
| Wake up early in the morning many days a week | 4.7 | 4.2 | 0.56 | 9.2 | 9.7 | 0.56 | 12.3 | 10.4 | 0.17 | 11.3 | 14.4 | 0.31 | 9.6 | 8.2 | <0.01 | 9.4 |
| Chronic disease limiting daily functions | 18.7 | 14.7 | <0.01 | 29.7 | 29.5 | 0.83 | 43.1 | 44.8 | 0.41 | 50.4 | 57.7 | 0.16 | 32.4 | 27.9 | <0.01 | 31.8 |
| **Symptoms in last 12 months** |  |  |  |  |  |  |  |  |  |  |  |  |  |  |  |  |
| Daily cough in periods (Q2) | 21.3 | 19.7 | 0.25 | 21.3 | 19.0 | 0.05 | 24.5 | 23.3 | 0.52 | 25.1 | 25.8 | 0.91 | 22.6 | 20.3 | <0.01 | 22.2 |
| Attacks of wheezing or breathlessness | 9.4 | 8.1 | 0.16 | 10.2 | 11.4 | 0.18 | 15.6 | 19.8 | <0.01 | 21.7 | 27.0 | 0.23 | 12.3 | 12.2 | 0.95 | 12.3 |
| Allergic rhinitis (Q2) | 25.5 | 26.7 | 0.41 | 19.3 | 20.5 | 0.30 | 15.2 | 17.3 | 0.14 | 14.4 | 14.8 | 0.89 | 18.7 | 21.8 | <0.01 | 19.2 |
| Heartburn (a lot) (Q2) | 5.8 | 4.3 | 0.04 | 7.9 | 5.6 | <0.01 | 7.0 | 4.3 | <0.01 | 7.1 | 4.4 | 0.34 | 7.2 | 4.8 | <0.01 | 6.8 |
| Headache (Q2) | 37.1 | 32.2 | <0.01 | 33.7 | 29.9 | <0.01 | 18.5 | 14.7 | 0.02 | 9.3 | 12.3 | 0.31 | 27.8 | 27.3 | 0.51 | 27.7 |
| Migraine (Q2) | 5.5 | 6.5 | 0.20 | 5.7 | 5.9 | 0.81 | 3.8 | 2.4 | 0.06 | 4.3 | 0 | 0.02 | 5.0 | 5.3 | 0.45 | 5.0 |
| Muscleoskeletal pain of more than 3 months | 33.2 | 19.2 | <0.01 | 46.5 | 36.1 | <0.01 | 49.3 | 42.0 | <0.01 | 43.8 | 50.9 | 0.19 | 45.1 | 31.6 | <0.01 | 42.9 |
| Urine incontinence (Q2) | 4.9 | 1.7 | <0.01 | 7.6 | 3.1 | <0.01 | 10.6 | 9.0 | 0.22 | 14.0 | 24.2 | <0.01 | 8.7 | 5.2 | <0.01 | 8.2 |
|  |  |  |  |  |  |  |  |  |  |  |  |  |  |  |  |  |
| **History of diseases** |  |  |  |  |  |  |  |  |  |  |  |  |  |  |  |  |
| Medication for arterial hypertension | 1.6 | 3.4 | <0.01 | 14.6 | 18.5 | <0.01 | 39.7 | 51.2 | <0.01 | 47.8 | 50.0 | 0.70 | 21.4 | 23.8 | <0.01 | 21.7 |
| Myocardial infarction | 0.1 | 0.3 | 0.17 | 2.3 | 3.2 | 0.04 | 10.3 | 17.8 | <0.01 | 20.3 | 28.9 | 0.04 | 5.2 | 6.9 | <0.01 | 5.4 |
| Angina pectoris | 0.3 | 1.0 | 0.02 | 1.8 | 2.5 | 0.10 | 9.6 | 15.0 | <0.01 | 20.9 | 27.9 | 0.08 | 4.8 | 5.9 | 0.01 | 5.0 |
| Cerebral insult | 0.6 | 0.6 | 1.00 | 1.4 | 1.7 | 0.24 | 5.7 | 8.0 | 0.02 | 10.4 | 23.6 | <0.01 | 3.0 | 3.7 | 0.03 | 3.1 |
| Renal disease | 1.0 | 2.2 | 0.03 | 2.0 | 3.2 | <0.01 | 3.8 | 6.1 | <0.01 | 5.4 | 6.2 | 0.68 | 2.5 | 3.8 | <0.01 | 2.7 |
| Asthma | 10.4 | 11.7 | 0.20 | 8.9 | 8.9 | 0.96 | 9.5 | 11.5 | 0.09 | 8.5 | 11.1 | 0.37 | 9.4 | 10.5 | 0.04 | 9.5 |
| COPD or chronic bronchitis | 1.1 | 2.1 | 0.04 | 2.3 | 4.2 | <0.01 | 6.1 | 9.7 | <0.01 | 7.4 | 15.0 | 0.01 | 3.4 | 5.3 | <0.01 | 3.7 |
| Diabetes | 0.8 | 1.2 | 0.17 | 3.4 | 6.4 | <0.01 | 9.1 | 16.5 | <0.01 | 9.2 | 12.8 | 0.23 | 4.9 | 6.6 | <0.01 | 5.2 |
| Cancer | 0.9 | 1.0 | 0.73 | 2.2 | 2.8 | 0.23 | 9.7 | 14.0 | <0.01 | 14.8 | 13.9 | 0.90 | 4.9 | 4.6 | 0.55 | 4.8 |
| Osteoporosis | 0.1 | 0.1 | 1.00 | 0.4 | 0.8 | 0.03 | 1.2 | 2.1 | 0.08 | 3.7 | 4.6 | 0.62 | 0.7 | 1.1 | 0.03 | 0.8 |
| Fibromyalgia | 0.2 | 0.9 | 0.02 | 0.8 | 1.4 | 0.05 | 1.1 | 1.6 | 0.09 | 1.1 | 1.9 | 0.35 | 0.8 | 1.3 | <0.01 | 0.9 |
| Arthrosis | 1.0 | 2.2 | 0.01 | 7.1 | 8.2 | 0.13 | 19.0 | 25.8 | <0.01 | 26.1 | 38.2 | <0.01 | 10.3 | 11.9 | 0.01 | 10.5 |
| Sought help for mental problem | 8.7 | 10.9 | 0.03 | 11.1 | 13.4 | 0.01 | 9.1 | 10.2 | 0.38 | 8.0 | 8.5 | 0.86 | 9.9 | 11.7 | <0.01 | 10.1 |
| Hyperthyreosis | 0.3 | 0.1 | 0.70 | 0.7 | 0.5 | 0.38 | 1.3 | 0.8 | 0.47 | 1.8 | 1.7 | 1.00 | 0.9 | 0.5 | 0.05 | 0.8 |
| Hypothyreosis | 0.7 | 0.7 | 1.00 | 2.0 | 1.6 | 0.40 | 3.2 | 2.8 | 0.64 | 4.9 | 3.3 | 0.64 | 2.3 | 1.8 | 0.06 | 2.2 |
|  |  |  |  |  |  |  |  |  |  |  |  |  |  |  |  |  |
| **Life style** |  |  |  |  |  |  |  |  |  |  |  |  |  |  |  |  |
| Never-smoker § | 58.6 | 56.6 | 0.23 | 45.2 | 37.4 | <0.01 | 34.9 | 23.5 | <0.01 | 34.3 | 22.4 | <0.01 | 44.3 | 41.1 | <0.01 | 43.8 |
| Ex-smoker | 14.2 | 16.9 | 0.02 | 31.3 | 31.6 | 0.86 | 50.9 | 51.2 | 0.91 | 62.7 | 59.5 | 0.49 | 35.2 | 31.1 | <0.01 | 34.6 |
| Occasional smoker | 16.7 | 13.6 | <0.01 | 8.8 | 8.2 | 0.46 | 6.2 | 4.8 | 0.13 | 10.3 | 1.6 | <0.01 | 9.7 | 9.2 | 0.38 | 9.6 |
| Daily smoker | 12.6 | 12.9 | 0.77 | 17.9 | 22.8 | <0.01 | 18.5 | 20.6 | 0.19 | 14.8 | 16.7 | 0.60 | 16.9 | 18.7 | <0.01 | 17.1 |
| Daily use of snuff | 23.3 | 22.7 | 0.76 | 16.6 | 19.9 | <0.01 | 5.1 | 4.2 | 0.30 | 4.0 | 6.9 | 0.13 | 13.8 | 17.0 | <0.01 | 14.3 |
| Alcohol > 2-3 times a week | 12.6 | 13.4 | 0.44 | 21.0 | 20.9 | 1.00 | 20.6 | 19.5 | 0.67 | 12.0 | 8.9 | 0.33 | 18.8 | 17.8 | 0.16 | 18.6 |
| Exercise > 2-3 times a week | 14.3 | 11.9 | 0.04 | 13.2 | 13.1 | 0.61 | 23.2 | 18.9 | 0.01 | 33.8 | 27.4 | 0.27 | 17.3 | 13.9 | <0.01 | 16.9 |

¤ Invited to QNP, persons having died or emigrated between HUNT3 and QNP are excluded.

^#^  Height and weight measured at the screening stations, self-reported in QNP

§ Questions on tobacco smoking; included in n if answered at least one of the smoking related questions

£ CONOR Mental Health Index (CONOR MHI) consists of 7 questions on mental distress with score 1-4. Mean score calculated and cut-off for dichotomization was > 2.15.
