# Supplementary material for: The HUNT study: participation is associated with survival and depends on socioeconomic status, diseases and symptoms
Source: BMC Med Res Methodol. 2012 Sep 14;12:143. doi: 10.1186/1471-2288-12-143 (PMC3512497; doi:10.1186/1471-2288-12-143)
Supplement: Additional file 2 — Table S2. Comparisons of anthropometrics (means) and percentages reporting symptoms and diseases between participants having answered questionnaire 1 (Q1) (27 758) or questionnaire 2 (Q2), and those having answered a shortened nonparticipation questionnaire (QNP) (n = 3241) among women stratified by age groups. [file 1471-2288-12-143-S2.doc]

Table 1B Comparisons of anthropometrics (means) and percentages reporting symptoms and diseases between participants having answered questionnaire 1 (Q1) (27 758) or questionnaire 2 (Q2), and those having answered a shortened nonparticipation questionnaire (QNP) (n=3241) among women. Questions from Q2 are marked

|  | Age groups | | | | | | | | | | | | | | | |
| --- | --- | --- | --- | --- | --- | --- | --- | --- | --- | --- | --- | --- | --- | --- | --- | --- |
| WOMEN | 20-39 years | | | 40-59 years | | | 60-79 years | | | 80 years + | | | Total | | | |
|  | Q1 | QNP | p | Q1 | QNP | p | Q1 | QNP | p | Q1 | QNP | p | Q1 | QNP | p | Q1+QNP |
|  |  |  |  |  |  |  |  |  |  |  |  |  |  |  |  |  |
| Number invited ¤ | 14 823 | 8119 |  | 16 939 | 5491 |  | 11 475 | 3160 |  | 4056 | 2234 |  | 47 293 | 19 004 |  |  |
| Number participated | 6664 | 1372 |  | 11438 | 1109 |  | 8217 | 562 |  | 1439 | 198 |  | 27 758 | 3241 |  | 30 999 |
| Percent of invited to HUNT3 | 45.0 | 9.3 |  | 67.5 | 6.5 |  | 71.6 | 4.9 |  | 35.5 | 4.9 |  | 58.7 | 6.9 |  | 65.6 |
|  |  |  |  |  |  |  |  |  |  |  |  |  |  |  |  |  |
| Height (cm) ^#^ | 166.8 | 167.3 | <0.01 | 165.7 | 166.7 | <0.01 | 162.3 | 164.5 | <0.01 | 157.6 | 160.7 | <0.01 | 164.6 | 166.3 | <0.01 | 164.7 |
| Weight (kg) ^#^ | 71.7 | 70.5 | <0.01 | 73.9 | 72.9 | 0.02 | 73.4 | 73.6 | 0.77 | 67.4 | 64.4 | <0.01 | 72.9 | 71.5 | <0.01 | 72.8 |
| BMI (kg/m^2^) ^#^ | 25.8 | 25.2 | <0.01 | 26.9 | 26.2 | <0.01 | 27.9 | 27.1 | <0.01 | 27.1 | 25.0 | <0.01 | 26.9 | 25.8 | <0.01 | 26.8 |
|  |  |  |  |  |  |  |  |  |  |  |  |  |  |  |  |  |
| **Health care utilisation** |  |  |  |  |  |  |  |  |  |  |  |  |  |  |  |  |
| General practitioner last 12 months | 81.4 | 81.6 | 0.90 | 81.1 | 85.1 | <0.01 | 88.5 | 89.5 | 0.48 | 90.6 | 90.4 | 0.90 | 83.9 | 84.6 | 0.30 | 83.9 |
| Hospitalized last 12 months | 15.2 | 20.1 | <0.01 | 9.4 | 16.8 | <0.01 | 13.3 | 23.0 | <0.01 | 17.6 | 30.1 | <0.01 | 12.4 | 20.1 | <0.01 | 13.1 |
|  |  |  |  |  |  |  |  |  |  |  |  |  |  |  |  |  |
| **Self reported health and mental distress** |  |  |  |  |  |  |  |  |  |  |  |  |  |  |  |  |
| Current health poor or very poor | 14.8 | 16.5 | 0.07 | 25.6 | 32.4 | <0.01 | 39.6 | 43.7 | 0.06 | 52.9 | 64.9 | <0.01 | 28.4 | 29.6 | 0.20 | 28.5 |
|  |  |  |  |  |  |  |  |  |  |  |  |  |  |  |  |  |
| Mental distress £ | 7.8 | 11.6 | <0.01 | 7.6 | 11.7 | <0.01 | 8.1 | 13.2 | <0.01 | 9.8 | 16.8 | 0.01 | 7.9 | 12.1 | <0.01 | 8.3 |
| Insomnia many evening a week | 9.9 | 12.9 | <0.01 | 14.3 | 17.5 | <0.01 | 18.3 | 20.8 | 0.14 | 17.9 | 16.6 | 0.69 | 14.8 | 16.2 | 0.03 | 15.0 |
| Wake up early in the morning many days a week | 5.7 | 5.7 | 1.00 | 12.4 | 15.3 | <0.01 | 15.3 | 16.1 | 0.58 | 18.8 | 18.3 | 0.92 | 12.2 | 11.9 | 0.66 | 12.1 |
| Chronic disease limiting daily functions | 19.8 | 21.0 | 0.30 | 33.7 | 38.6 | <0.01 | 43.7 | 46.4 | 0.24 | 50.2 | 65.7 | <0.01 | 33.9 | 33.9 | 1.00 | 33.9 |
|  |  |  |  |  |  |  |  |  |  |  |  |  |  |  |  |  |
| **Symptoms in last 12 months** |  |  |  |  |  |  |  |  |  |  |  |  |  |  |  |  |
| Daily cough in periods (Q2) | 20.5 | 19.3 | 0.38 | 17.7 | 21.0 | <0.01 | 20.2 | 23.5 | 0.07 | 18.8 | 20.7 | 0.56 | 19.1 | 20.7 | 0.04 | 19.3 |
| Attacks of wheezing or breathlessness | 9.8 | 7.9 | 0.03 | 11.2 | 11.7 | 0.62 | 15.6 | 16.0 | 0.80 | 16.1 | 21.3 | 0.10 | 12.4 | 11.3 | 0.09 | 12.3 |
| Allergic rhinitis (Q2) | 29.1 | 27.4 | 0.26 | 24.0 | 25.7 | 0.22 | 18.9 | 23.1 | 0.03 | 14.3 | 16.1 | 0.50 | 23.1 | 25.3 | <0.01 | 23.4 |
| Heartburn (a lot) (Q2) | 5.0 | 2.9 | <0.01 | 6.7 | 5.8 | 0.26 | 9.3 | 5.6 | <0.01 | 7.7 | 3.9 | 0.04 | 7.1 | 4.4 | <0.01 | 6.7 |
| Headache (Q2) | 57.1 | 52.7 | <0.01 | 48.0 | 47.1 | 0.56 | 27.5 | 28.4 | 0.68 | 17.1 | 21.3 | 0.18 | 42.1 | 44.7 | <0.01 | 42.4 |
| Migraine (Q2) | 13.3 | 14.5 | 0.24 | 13.3 | 13.8 | 0.68 | 6.5 | 4.2 | 0.03 | 6.4 | 3.3 | 0.13 | 10.9 | 11.8 | 0.09 | 11.0 |
| Muscleoskeletal pain of more than 3 months | 38.5 | 26.8 | <0.01 | 58.0 | 49.5 | <0.01 | 61.3 | 57.7 | 0.11 | 54.6 | 64.3 | 0.02 | 54.6 | 41.9 | <0.01 | 53.0 |
| Urine incontinence (Q2) | 21.8 | 14.9 | <0.01 | 27.8 | 17.4 | <0.01 | 27.8 | 24.4 | 0.09 | 34.9 | 44.2 | 0.01 | 26.8 | 20.6 | <0.01 | 26.1 |
|  |  |  |  |  |  |  |  |  |  |  |  |  |  |  |  |  |
| **History of diseases** |  |  |  |  |  |  |  |  |  |  |  |  |  |  |  |  |
|  |  |  |  |  |  |  |  |  |  |  |  |  |  |  |  |  |
| Medication for arterial hypertension | 2.2 | 3.1 | 0.18 | 13.5 | 17.4 | 0.01 | 39.4 | 54.7 | <0.01 | 52.0 | 62.7 | <0.01 | 20.5 | 24.9 | <0.01 | 20.8 |
| Myocardial infarction | 0 | 0.1 | 0.17 | 0.5 | 1.1 | 0.01 | 3.2 | 7.8 | <0.01 | 8.6 | 16.1 | <0.01 | 1.6 | 3.1 | <0.01 | 1.7 |
| Angina pectoris | 0.2 | 0.1 | 1.00 | 0.6 | 1.1 | 0.04 | 4.9 | 7.3 | 0.03 | 15.2 | 17.1 | 0.57 | 2.5 | 3.2 | 0.04 |  |
| Cerebral insult | 0.2 | 1.2 | <0.01 | 1.2 | 1.9 | 0.06 | 4.0 | 7.0 | <0.01 | 9.4 | 12.4 | 0.21 | 2.2 | 3.5 | <0.01 | 2.3 |
| Renal disease | 1.4 | 1.8 | 0.46 | 2.5 | 3.5 | 0.07 | 3.2 | 6.9 | <0.01 | 4.2 | 4.7 | 0.85 | 2.6 | 3.9 | <0.01 | 2.7 |
| Asthma | 12.2 | 12.0 | 0.89 | 9.5 | 9.8 | 0.70 | 10.5 | 11.9 | 0.31 | 8.6 | 6.4 | 0.38 | 10.4 | 10.9 | 0.39 | 10.4 |
| COPD or chronic bronchitis | 1.4 | 2.0 | 0.18 | 2.6 | 3.2 | 0.28 | 5.4 | 6.9 | 0.19 | 4.8 | 6.4 | 0.35. | 3.3 | 3.9 | 0.14 | 3.3 |
| Diabetes | 0.9 | 2.0 | <0.01 | 2.3 | 4.9 | <0.01 | 7.1 | 14.6 | <0.01 | 9.7 | 13.4 | 0.13 | 3.8 | 5.7 | <0.01 | 4.0 |
| Cancer | 1.0 | 0.8 | 0.75 | 4.0 | 5.3 | <0.01 | 10.1 | 13.1 | 0.03 | 13.4 | 17.3 | 0.16 | 5.6 | 5.6 | 0.94 | 5.6 |
| Osteoporosis | 0.2 | 0.4 | 0.41 | 1.8 | 2.3 | 0.24 | 11.7 | 15.2 | 0.02 | 21.3 | 22.5 | 0.71 | 5.3 | 5.9 | 0.20 | 5.3 |
| Fibromyalgia | 1.8 | 2.6 | 0.13 | 6.9 | 8.6 | 0.04 | 9.5 | 12.5 | 0.04 | 4.6 | 5.0 | 0.71 | 6.3 | 7.4 | 0.03 | 6.4 |
| Arthrosis | 1.3 | 3.4 | <0.01 | 13.7 | 17.3 | 0.01 | 36.5 | 43.2 | <0.01 | 46.4 | 53.9 | 0.06 | 18.8 | 21.4 | <0.01 | 19.0 |
| Sought help for mental problem | 16.8 | 16.5 | 0.84 | 18.5 | 25.5 | <0.01 | 15.6 | 20.9 | <0.01 | 10.8 | 19.7 | <0.01 | 16.9 | 20.6 | <0.01 | 17.7 |
| Hyperthyreosis | 1.3 | 0.7 | 0.34 | 2.7 | 2.8 | 0.85 | 3.8 | 5.9 | 0.04 | 3.8 | 5.8 | 0.26 | 2.8 | 3.0 | 0.56 | 2.8 |
| Hypothyreosis | 4.0 | 4.7 | 0.40 | 8.4 | 9.7 | 0.15 | 13.7 | 17.1 | 0.04 | 13.1 | 12.2 | 0.90 | 9.3 | 10.0 | 0.30 | 9.4 |
|  |  |  |  |  |  |  |  |  |  |  |  |  |  |  |  |  |
| **Life style** |  |  |  |  |  |  |  |  |  |  |  |  |  |  |  |  |
| Never-smoker § | 54.9 | 52.3 | 0.09 | 38.4 | 34.8 | 0.02 | 49.4 | 36.0 | <0.01 | 73.3 | 66.8 | 0.07 | 47.3 | 44.4 | <0.01 | 47.0 |
| Ex-smoker | 17.0 | 18.3 | 0.25 | 30.6 | 30.0 | 0.64 | 32.6 | 38.4 | <0.01 | 21.2 | 23.8 | 0.45 | 27.4 | 26.1 | 0.13 | 27.3 |
| Occasional smoker | 12.7 | 12.9 | 0.82 | 8.6 | 8.3 | 0.73 | 7.8 | 4.4 | <0.01 | 8.2 | 4.1 | 0.06 | 9.4 | 9.3 | 0.92 | 9.4 |
| Daily smoker | 17.0 | 16.5 | 0.69 | 25.3 | 26.9 | 0.23 | 19.1 | 21.1 | 0.24 | 8.5 | 5.2 | 0.16 | 20.6 | 20.2 | 0.59 | 20.6 |
| Daily use of snuff | 2.8 | 3.1 | 0.46 | 1.3 | 1.8 | 0.18 | 0 | 0.4 | 0.03 | 0.1 | 0 | 1.00 | 1.2 | 1.9 | <0.01 | 1.3 |
| Alcohol > 2-3 times a week | 6.1 | 5.3 | 0.33 | 15.9 | 13.8 | 0.07 | 12.4 | 12.6 | 0.90 | 5.4 | 1.9 | 0.04 | 12.0 | 9.5 | <0.01 | 11.3 |
| Exercise> 2-3 times a week | 16.6 | 12.0 | <0.01 | 19.0 | 14.7 | <0.01 | 24.5 | 16.3 | <0.01 | 31.0 | 20.4 | 0.03 | 20.5 | 14.0 | <0.01 | 19.8 |

¤ Invited to QNP, persons having died or emigrated between HUNT3 and QNP are excluded.

^#^  Height and weight measured at the screening stations, self-reported in QNP

§ Questions on tobacco smoking; included in n if answered at least one of the smoking related questions

£ CONOR Mental Health Index (CONOR MHI) consists of 7 questions on mental distress with score 1-4. Mean score calculated and cut-off for dichotomization was > 2.15.
